# L’esthésioneuroblastome pédiatrique: une lésion maligne exceptionnelle (à propos d’un cas et revue de la littérature)

**DOI:** 10.11604/pamj.2018.31.144.16807

**Published:** 2018-10-25

**Authors:** Mehdi Borni, Brahim Kammoun, Fatma Kolsi, Mohamed Zaher Boudawara

**Affiliations:** 1 Service de Neurochirurgie, CHU Habib Bourguiba de Sfax, Tunisie

**Keywords:** Esthésioneuroblastome, exérèse, chimiothérapie, radiothérapie, Esthesioneuroblastoma, excision, chemotherapy, radiotherapy

## Abstract

L'esthésioneuroblastome (ENB) est une tumeur maligne rare représentant 3% des cancers des cavités naso-sinusiennes; son origine se situe au niveau de l'épithélium olfactif. Elle touche généralement des sujets de 30 à 50 ans. Elle est exceptionnelle chez l'enfant. Le diagnostic est souvent tardif, du fait du caractère longtemps confiné de la tumeur et le pronostic dépend des extensions locorégionales (notamment cérébrales et orbitaires). Nous rapportons un cas de cette affection à localisation sphénoïdale chez un sujet de 03 ans découverte suite à une cécité d'installation rapide dont on discute les particularités cliniques, radiologiques, anatomopathologiques, thérapeutiques et pronostique tout en insistant sur la précocité de la prise en charge conditionnant le pronostic qui restait malheureusement toujours péjoratif du fait du taux de récidives assez élevé ainsi que la survenue de métastases à distance, notamment pulmonaires et osseuses.

## Abstract (en)

Esthesioneuroblastoma (ENB) is a rare malignant tumor accounting for 3% of all sinonasal cancers. It arises from the olfactory epithelium and usually affects subjects aged 30-50 years. It is uncommon in children. It is often diagnosed late because tumor remains confined to its original site for long and prognosis depends on locoregional extension (in particular to the brain and the orbital regions). We report the case of a 3-year old child with sphenoidal esthesioneuroblastoma discovered after early onset blindness. This study aims to highlight the clinical, radiological, anatomopathological, therapeutic and prognostic peculiarities of this disease while insisting on the importance of early diagnosis affecting prognosis. Unfortunately, diagnosis is still pejorative due to high recidivism rates as well as to the occurrence of distant metastases (in particular lung and bone metastases).

## Introduction

L'esthésioneuroblastome(ENB) est une tumeur maligne rare représentant 3% des cancers des cavités naso-sinusiennes; son origine se situe au niveau de l'épithélium olfactif. Son incidence exacte est difficile à établir du fait d'un diagnostic histologique de certitude complexe. Moins de 1000 cas dans le monde ont été publiés durant les 20 dernières années. Elle touche généralement des sujets de 30 à 50 ans. Elle est exceptionnelle chez l'enfant. Le diagnostic est souvent tardif, du fait du caractère longtemps confiné de la tumeur et le pronostic dépend des extensions locorégionales (notamment cérébrales et orbitaires).

## Patient et observation

**Figure 1:**
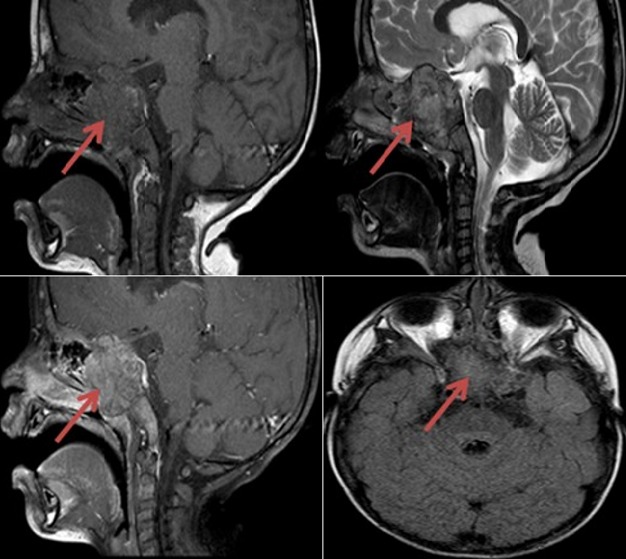
IRM en séquence T1, T2, avec gadolinium et T2 Flair montrant la lésion osseuse de remplacement médullaire en hypo signal T1 et hyper signal T2, avec des spots spontanément hyper intenses refoulant le chiasma optique et s’étend vers la fissure orbitaire engainant les deux nerfs optiques. Latéralement, elle envahit le sinus caverneux gauche. En avant, elle envahit les cellules ethmoïdales et les fosses nasales refoulant la lame orbitaire interne droite

Il s'agit d'un patient âgé de 03 ans jusque-là sans antécédents qui est admis pour cécité bilatérale d'installation rapide. Son examen neurologique est sans particularités à part la cécité et l'abolition des réflexes photomoteurs aux deux yeux. L'IRM cérébrale et du massif facial (Figure 1) a objectivé une volumineuse lésion centrée sur le corps du sphénoïde, consistant en une lésion osseuse de remplacement médullaire en hypo signal T1 et hyper signal T2, avec des spots spontanément hyper intenses. En bas, l'extension semble respecter la synchondrose sphéno-occipitale. En haut elle réalise une soufflure des crinoïdes et du jugum refoulant le chiasma optique et s'étend vers la fissure orbitaire engainant les deux nerfs optiques. Latéralement, il envahit le sinus caverneux gauche. En avant, il envahit les cellules ethmoïdales et les fosses nasales refoulant la lame orbitaire interne droite associé à une composante intra orbitaire extra conale refoulant le droit interne et respectant la graisse. Le patient a donc subi une exérèse large par voie fronto-ptérionale d'une lésion grisâtre moyennement hémorragique comportant des foyers de saignement ancien. La base du crâne est devenue molle au niveau du jugum sphénoïdal ayant perdu sa consistance osseuse.

## Discussion

L'ENB ou neuroblastome olfactif est une tumeur rare (1000 cas dans la littérature), décrite pour la première fois par Berger en 1924 [1]. Elle concerne préférentiellement l'homme, et survient le plus souvent au cours de la deuxième et de la troisième décade [2]. Les manifestations cliniques révélatrices ne peuvent revendiquer aucun signe propre, d'autant plus que plusieurs symptômes peuvent s'associer. Elles témoignent souvent d'un processus extensif intra nasal et sont donc dominées par des signes rhinologiques. Les signes neurologiques sont fréquents et les manifestations ophtalmologiques notamment l'exophtalmie et la diplopie traduisent l'extension tumorale au niveau orbitaire [3]. L'examen ORL doit obligatoirement comporter une endoscopie nasale au tube rigide, qui permettra de mettre en évidence la tumeur sous la forme d'une masse bourgeonnante et de faire des biopsies. L'otoscopie peut mettre en évidence une otite séro-muqueuse. Celle des aires ganglionnaires cervicales, à la recherche d'adénopathies, est également indispensable. Celles-ci sont retrouvées dans 10 à 28% des cas. Le siège des métastases est variable selon les auteurs, mais il semble que la localisation cérébrale est la plus fréquente. Elle est expliquée par la proximité de la lame criblée qui réalise une voie directe d'extension vers les méninges [4]. Cette tumeur prend son origine au niveau de l'épithélium olfactif, plus particulièrement au niveau de la lame criblée, du tiers supérieur du septum et du cornet supérieur. Elle reproduit la structure embryonnaire de la placode olfactive. Selon le degré de différenciation des cellules, on en distingue 3 types: L'esthésioneuro épithéliome, l'esthésioneurocytome et l'ENB. Sur le plan histologique, l'ENB apparaît comme une prolifération de cellules peu différenciées, en nappes diffuses ou lobules séparés par des cloisons conjonctivo-vasculaires. Des rosettes de typeHomer-Wright ou Flexner-Winstersteiner peuvent être présentes. En immuno-histochimie, il existe une positivité pour certains marqueurs, notamment neuroendocrines (NSE, synaptophysine, neurofilaments, CD56). En microscopie à balayage électronique, on peut noter la présence de granules denses endocrinoïdes intra cytoplasmiques. Les principaux diagnostics différentiels généralement évoqués sont le carcinome sinonasalin différencié, le carcinome neuroendocrine sinonasal, le carcinome à petites cellules, l'adénome pituitaire, le mélanome, le lymphome, et le rhabdomyosarcome [5]. La TDM et l'IRM jouent un rôle très important dans le bilan d'extension de ces tumeurs. Leur aspect est relativement aspécifique, hétérogène, apparaissant isodense ou légèrement hyperdense, avec des images de nécrose disséminée et des images kystiques en périphérie. En IRM, ces tumeurs apparaissent en hyposignal en pondération T1 et en hypersignal hétérogène en T2. Dans ces deux modalités, les tumeurs se rehaussent de façon hétérogène après injection de produit de contraste [6]. Le scanner et l'IRM évaluent aussi l'importance de l'étendue tumorale et permettent la classification de Kadish: stade A: tumeur localisée à la fosse nasale; stade B: tumeur naso-sinusienne et stade C: tumeur étendue au-delà des cavités naso-sinusiennes [7]. Un bilan d'imagerie complet est crucial pour le bilan d'extension initial de la tumeur et la mise en évidence d'une éventuelle extension intracrânienne. Plus récemment, l'imagerie nucléaire, avec la TEP-scanographie au (18F)-fluorodésoxyglucose (FDG) a également démontré son intérêt, principalement dans le diagnostic des extensions à distance ou des récidives [8]. Le traitement de l'ENB est résolument multidisciplinaire, repose sur l'association chirurgie-radiothérapie, qui offrirait selon certains le meilleur pronostic [9]. Plusieurs voies d'abord sont possibles. Les résections endoscopiques sont réservées aux petites tumeurs, facilement contrôlables par voie endonasale. Les tumeurs plus évoluées seront abordées par voie para-latéro nasale, plus ou moins combinée à une incision bicoronale. Cette intervention est menée, si besoin, par une double équipe ORL et neurochirurgicale. Le curage ganglionnaire cervical n'est indiqué qu'en cas d'adénopathies palpables ou mises en évidence à l'imagerie. L'irradiation post-opératoire doit toujours être entreprise dans les tumeurs catégorisées au stade A et B du fait de taux élevé de récidive locale après chirurgie exclusive [10]. Pour les ENB étendues ou disséminés (stade C), la chimiothérapie doit être utilisée en première intention avant d'entreprendre la thérapeutique radio chirurgicale. Elle permet d'atteindre, selon les auteurs, 75% de survie à 2 ans [10]. L'évolution de l'ENB est marquée par des récidives fréquentes, elles surviennent dans 2/3 des cas; le délai d'apparition est de 18 mois environ [11]. Les métastases ganglionnaires et à distance sont possibles, et leur fréquence varie entre 18 et 62% [12].

## Conclusion

L'ENB est une tumeur maligne rare, et plus encore dans la population pédiatrique. Son développement dans des structures confinées explique son diagnostic tardif et l'extension fréquente aux organes de voisinage. Son traitement est délicat en raison de sa localisation. Mais le développement des nouvelles techniques de radiothérapie et de nouvelles drogues anticancéreuses devrait permettre une prise en charge moins agressive, et donc une meilleure qualité de vie tout en améliorant le pronostic.

## Conflits d’intérêts

Les auteurs ne déclarent aucun conflit d’intérêts.
